# Prevalence of Spirometry Testing among Patients with Asthma and COPD in German General Practices

**DOI:** 10.3390/healthcare10122570

**Published:** 2022-12-18

**Authors:** Annika Härtel, Maximilian Peters, Karel Kostev

**Affiliations:** 1 Real World Evidence, IQVIA, 60549 Frankfurt, Germany; 2 Epidemiology, IQVIA, 60549 Frankfurt, Germany; 3 University Hospital, Philipps-University Marburg, 35039 Marburg, Germany

**Keywords:** asthma, COPD, spirometry, general practice, lung function test

## Abstract

The goal of this study was to estimate the prevalence of spirometry testing among patients with asthma and chronic obstructive pulmonary disease (COPD) in general practices (GPs) in Germany. This retrospective cross-sectional study was based on data from the Disease Analyzer database (IQVIA), This retrospective cross-sectional study included all patients with at least one confirmed asthma or COPD diagnosis in one of those 50 general practices in Germany between January 2020, and January 2021, as well as at least one visit to these general practices between January 2021, and January 2022. The main outcomes of the study aimed to ascertain the proportion of spirometry testing among asthma and COPD patients between January 2021, and January 2022, overall, and separately, in men, women, six age groups (≤30, 31–40, 41–50, 51–60, 61, 70, >70), and patients who received at least one prescription of anti-asthma or anti-COPD drugs. This study included 8835 patients with asthma only, 5597 with COPD only, and 1897 with both asthma and COPD diagnoses. Of these, 27.2% of COPD patients, 7% of asthma patients, and 54.7% of asthma + COPD patients, received spirometry testing during the study period. Among COPD and asthma + COPD patients, the prevalence of spirometry testing was much higher in women than in men (COPD: 31.6% vs. 23.2%; asthma + COPD: 59.6% vs. 46.3%) and much higher in treated than in non-treated patients (COPD: 31.7% vs. 15.0%; asthma + COPD: 57.5% vs. 27.8%). The prevalence of spirometry testing was relatively low among COPD and asthma patients followed in GP practices, but usually higher in female patients, treated patients, and patients suffering from both asthma and COPD.

## 1. Introduction

Spirometry is a common test used to diagnose several conditions that affect breathing or monitor treatment for chronic lung diseases, including asthma and chronic obstructive pulmonary disease (COPD) [1]. Spirometry plays a very important role in clinical practice, as this test can assess pulmonary function of patients and enable measuring the effect of a disease on lung function, as well as prognosticating many pulmonary conditions [2] The role of spirometry is additionally important in the COVID-19 era as post-infection COVID-19 patients often show impaired lung function [3,4].

In primary care practices, spirometry can prevent wait times for testing in hospital laboratories and provide more timely data to physicians [2]. In 2017, Chapman et al. reported that 57% of physicians in Germany used spirometry in asthma patients and this proportion was higher than in France, Austria, Canada, China, and Japan [5]. Burkhardt and Pankow reported that 80% of primary care physicians’ offices in Germany were equipped with spirometers [6]. At the same time, Heinmüller et al. reported that, for Germany in 2020, of 2568 patients with COPD, 29% had had a spirometry test within the previous year [7].

However, no data have yet been published on the prevalence of spirometry testing in general practices in different patient cohorts based on demographic and clinical characteristics. This study aimed to close this gap.

## 2. Materials and Methods

This retrospective cross-sectional study was based on data from the Disease Analyzer database (IQVIA), which contains drug prescriptions, diagnoses, and basic medical and demographic data obtained directly, and in anonymous format, from computer systems used in the practices of general practitioners and specialists [8]. The database covers approximately 3% of all outpatient practices in Germany. It has previously been shown that the panel of practices included in the Disease Analyzer database is representative of general and specialized practices in Germany [8]. Finally, this database has already been used in previous studies focusing on chronic lower respiratory diseases [9,10].

Spirometry test values have been available in the database since January 2021, if practices connect the spirometry test device to their computer system. This is the case for 50 of the general practices in the Disease Analyzer. These values included forced expiratory volume (FEV1), forced vital capacity (FVC), and peak expiratory flow (PEF), as well as further values.

This study included all patients with at least one confirmed asthma (ICD-10: J45) or COPD (ICD-10: J44) diagnosis in one of the 50 general practices in the study in Germany between January 2020, and January 2021, as well as at least one visit to these general practices between January 2021, and January 2022. The main outcome of the study aimed to ascertain the proportion of spirometry testing among asthma and COPD patients between January 2021, and January 2022, overall, and separately, in men, women, six age groups (≤30, 31–40, 41–50, 51–60, 61, 70, >70), and patients who received at least one prescription of anti-asthma or anti-COPD drugs (ATC: R03F). Differences in the sample characteristics and diagnosis prevalence between those with and those without spirometry measures were tested using chi-squared tests for categorical variables and Wilcoxon tests for age.

*P*-values of <0.05 were considered statistically significant. Analyses were carried out using SAS version 9.4 (SAS Institute, Cary, NC, USA).

## 3. Results

The present study included 16,329 individuals with at least one visit to one of 50 general practices between January 2021, and January 2022, and at least one diagnosis of asthma or COPD between January 2020, and January 2021. Of the patients included, 8835 had asthma only, 5597 had COPD only, and 1897 had both asthma and COPD diagnoses.

During the study period, 1524 (27.2%) of the COPD patients, 615 (7.0%) of the asthma patients, and 1038 (54.7%) of the asthma and COPD patients received spirometry testing.

Among COPD patients, average FEV1 was 1.9 (SD: 0.8) L, FVC 2.7 (SD: 1.0) L, and PEF 5.0 (SD: 2.0) L/s. Among asthma patients, average FEV1 was 2.5 (SD: 1.0) L, FVC 3.2 (SD: 1.2) L, and PEF 5.7 (SD: 2.2) L/s. Among patients with both COPD and asthma, average FEV1 was 2.4 (SD: 0.9) L, FVC 3.1 (SD: 1.0) L, and PEF 6.1 (SD: 2.21) L/s.

**Figure 1:**
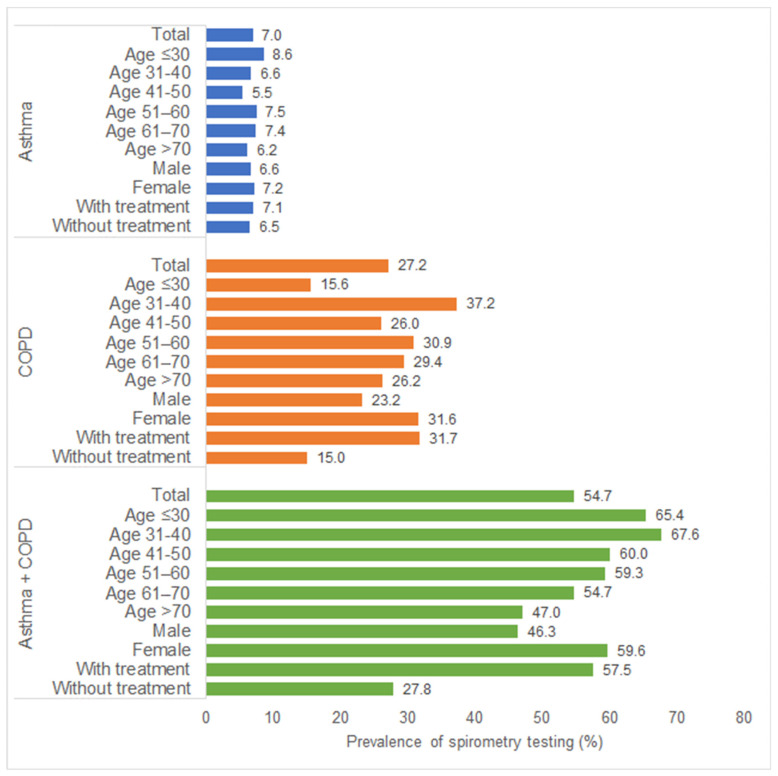
Prevalence of spirometry testing among asthma and COPD patients in general practices in Germany.

Among asthma patients, no relevant differences were observed between age groups, women and men, and treated and non-treated patients in terms of spirometry testing prevalence. Among COPD and asthma + COPD patients, the prevalence of spirometry testing was much higher in women than in men (COPD: 31.6% vs. 23.2%; asthma + COPD: 59.6% vs. 46.3%) and much higher in treated than in non-treated patients (COPD: 31.7% vs. 15.0%; asthma + COPD: 57.5% vs. 27.8%) (Figure 1).

The basic characteristics of study patients are displayed in Table 1. Among COPD and asthma + COPD patients, patients with spirometry testing were significantly younger (COPD: 65.3 vs. 67.7 years; asthma + COPD: 59.0 vs. 64.8 years) and significantly more were women (COPD: 56.1% vs. 45.8%; asthma + COPD: 69.4% vs. 56.7%) (Table 1).

## 4. Discussion

The indications for spirometry testing include diagnoses of respiratory diseases (e.g., COPD, bronchial asthma) and monitoring of the course and therapy of bronchopulmonary diseases [11].

In terms of the prevalence of spirometry testing among COPD patients in Germany, our results (27%) were in line with those of Heinmüller et al., who reported a prevalence of 29% 5. In a study performed in primary care practices in the USA, the yearly spirometry prevalence in COPD patients was 27% [12]. In order to determine if this relatively low prevalence indicated suboptimal COPD care in general practices, it was important to consider the role of pneumologists in COPD patient care. Based on the data from the same Disease Analyzer database used for the present study, one GP practice had 55 patients with COPD diagnoses in 2021, while one pneumologist practice had 618 COPD patients during the same period. Due to the much higher number of GP practices than pneumologist practices in Germany, more than 70% of COPD patients were treated in GP practices. However, it could be assumed that there was a difference in COPD severity between those patients treated by GPs and those treated by pneumologists.

Rubio et al. analyzed the use of spirometry in individuals with chronic respiratory symptoms in Spain. Of the individuals who saw a doctor, 69% underwent spirometry, with those seen by a pulmonologist being 6 times more likely to undergo spirometry. On the other hand, the proportion of individuals undergoing spirometry in the study increased significantly between 2005 and 2021 [13]. Although the study was carried out in Spain, it underlined the role of pulmonologists in ensuring patients undergo spirometry, which might explain why a significant proportion of patients visiting GPs do not receive spirometry and are instead referred to pulmonologists.

Hausen expressed the opinion that early diagnosis of COPD is the domain of the GP, although COPD is too often not discovered until it is at an advanced stage [14].

In our study, the prevalence of spirometry testing was higher in women than in men. This difference might be due to the fact that women with COPD are more likely to experience dyspnea and have a more severe course of the disease than men [15]. As monitoring of the therapy of bronchopulmonary diseases is one of the indications for spirometry [11], this might explain why patients who received therapy in our study had a higher spirometry testing prevalence than those who did not receive therapy in GP practices.

The role of spirometry in the diagnosis of asthma is as prominent as in diagnosing COPD. The only limitation is that initial spirometry often fails to show obstruction, as pulmonary function values may appear normal if measured during a symptom-free interval. However, this cannot rule out the possibility of disease [16,17]. In the investigation of the diagnostic accuracy of spirometry in primary care, Schneider et al. reported that the sensitivity of the testing in diagnosing COPD was 92% compared to just 29% in diagnosing asthma [18]. Furthermore, the first symptoms of asthma often occur in children [19] and in such cases, pediatricians rather than GPS are responsible for patient support. NICE guidelines recommend performing spirometry for the monitoring of asthma at least 3 or 6 months after the beginning of therapy and every 1–2 years thereafter [20]. In our study, only 7% of asthma patients underwent spirometry at least once during the study period. Again, in Germany, both GPs and pneumologists treat asthma patients. In 2021, a single GP practice admitted 67 asthma patients, while the average number of asthma patients per pneumologist practice during the same year was 898. As there are approximately 60 times more private GP practices than pneumologist practices in Germany, 80% of asthma patients are treated by GPs. Based on this information, it can be assumed that spirometry testing is underused in GP practices. By contrast, 55% of asthma patients who additionally had COPD underwent spirometry.

In a study performed in Italy, 55% of asthma patients had never undergone spirometry and the average time since a patient’s last spirometry was about 47 months [21]. Another study from Sweden found that 33% of asthma patients were tested using spirometry or Peak Expiratory Flow (PEF) during their initial visits and 60% during their 24-month follow-up [22].

The strengths of this study included the high number of general practices and outpatients available for analysis, as well as the use of real-world data. Nonetheless, the results of this study should be interpreted in light of several limitations.

First, asthma and COPD diagnoses relied solely on ICD codes used in general physician practices, and no information was available regarding the procedure used to diagnose these diseases. Second, no data were available on the socioeconomic and lifestyle-related risk factors of patients. Third, in the database used, each patient could only be followed up by one doctor, and no information was available as to whether study patients had undergone spirometry testing in pneumologist practices or hospitals, which could significantly increase the total prevalence of spirometry testing. This could also be the reason why the proportion of treated patients was low in our study. Fourth, no information on spirometer types (i.e., body plethysmograph, pneumotachometer, electronic and incentive spirometer, peak flow meter) was available in the database. Finally, the study was conducted in Germany and based on data from GPs, and its findings may not be transferable to other populations, other specialties, and other countries.

As GPs are primarily responsible for the support of asthma and COPD patients in Germany, and there is a lack of specialist practices in Germany, an increase in the use of spirometry by primary care physicians should be expected in the next few years.

## 5. Conclusions

In conclusion, the prevalence of spirometry testing was relatively low among COPD and asthma patients followed in GP practices, but usually higher in female patients, patients receiving treatment, and patients suffering from both asthma and COPD.

## Figures and Tables

**Table 1:** Age and sex characteristics of asthma and COPD patients with and without spirometry testing.

Variable	All Patients	Patients with Spirometry Testing	Patients without Spirometry Testing	*p*-Values
Asthma				
Age (mean, SD)	51.5 (19.7)	50.3 (20.4)	51.6 (19.7)	0.164
Female (%)	60.4	62.6	60.2	0.239
COPD				
Age (mean, SD)	67.0 (14.0)	65.3 (14.8)	67.7 (13.6)	<0001
Female (%)	48.8	56.1	45.8	<0.001
Asthma +COPD				
Age (mean, SD)	61.6 (13.3)	59.0 (16.3)	64.8 (15.8)	<0001
Female (%)	63.7	69.4	56.7	<0.001

## Data Availability

The data that support the findings of this study are available from the corresponding author upon reasonable request.
